# Single cell transcriptomics comes of age

**DOI:** 10.1038/s41467-020-18158-5

**Published:** 2020-08-27

**Authors:** Sarah Aldridge, Sarah A. Teichmann

**Affiliations:** 1 Wellcome Sanger Institute, Wellcome Genome Campus, Hinxton, Cambridge, CB1 1SA UK; 2 Theory of Condensed Matter Group, Cavendish Laboratory/Department of Physics, University of Cambridge, JJ Thomson Ave, Cambridge, CB3 OHE UK

**Keywords:** Gene expression analysis, Computational biology and bioinformatics

## Abstract

Single cell transcriptomics technologies have vast potential in advancing our understanding of biology and disease. Here, Sarah Aldridge and Sarah Teichmann review the last decade of technological advancements in single-cell transcriptomics and highlight some of the recent discoveries enabled by this technology.

The past decade has seen a revolution in single-cell transcriptomics. Here we describe advances made and the transformation that this powerful approach has on our ability to build detailed cellular maps of tissues, gaining unique insights in health and disease.

## Development of single-cell transcriptomics technologies over the past decade

The history of single-cell transcriptomics connects to precursor methods, such as single-cell qPCR, performed for the first time on a small number of genes by several groups in the early 90 s (e.g. refs. ^1,2^). To our knowledge, the first example of bona fide single-cell transcriptomics is the study of a handful of mouse primordial germ cells by Tang et al. in 2009^3^. While this work was motivated by the sparsity of very rare cells that arise during murine development, key to driving forwards single-cell transcriptomics has been the scaling of technologies to profile large numbers of cells in parallel. The ability to perform unbiased single-cell transcriptome-wide analysis coupled with exponential scaling in cell numbers that can be analysed simultaneously has almost followed a “Moore’s Law” of single-cell genomics.

**Fig. 1:**
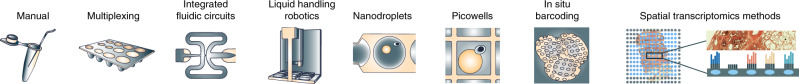
Development of single-cell technologies. Significant technology developments have enabled the profiling of large numbers of cells in parallel by single-cell transcriptomics. Initial manual methods allowed single-cell transcriptomic analysis on only a few cells. The development of integrated fluidic circuits and the introduction of liquid handling robots into the process brought cell numbers to several thousand and then further increased with nanodroplet and picowell technologies. The introduction of in situ barcoding has increased throughput even further to hundreds of thousands of cells with the latest developments in spatial methods integrating the spatial location of transcriptomic information within tissue sections.

Following Tang et al., other single-cell transcriptomics protocols were developed including tag sequencing methods such as STRT-seq, and CEL-seq, MARS-seq and full length protocols such as SMART-seq/SMART-seq2, as well as other methods (Fig. 1). These protocols differed in their amplification technology and transcript coverage, as well as in the extent of robotisation of liquid handling in plates. This was followed by development of nanodroplets, picowell technologies and in situ barcoding which have made it possible to sequence tens of thousands of cells in parallel (reviewed in ref. ^4^). In recent years multi-modal single-cell methodologies that measure and integrate genomics readouts from different molecules (RNA, DNA and protein)^5^, enabled dissection of the complex regulatory and cell-cell communication networks that drive cell identity to a greater degree than ever before.

Computational methods for low-level processing, quality control and ultimately sophisticated data interpretation were developed rapidly alongside the first single-cell transcriptomics data sets^6^. These methods are crucial for defining cell states and types from data, revealing marker genes and identifying developmental trajectories that relate cells to each other. The more recent multi-omics methods are catalysing new computational methods^5^ as are spatial methods discussed below.

Methods for whole transcriptome spatial mapping, characterisation of gene expression profiles whilst retaining information on spatial context of expression, are fast emerging. smFISH has been used for a number of years for analysing gene expression whilst retaining spatial information in tissue. Methodologies based on this include low-plex that analyse only a small number of genes in parallel, up to highly multiplexed that interrogate hundreds or thousands of mRNA targets in parallel, for example MERFISH, in situ sequencing, seqFISH, that have emerged over the past five years or so. Spatial transcriptomics methods, first developed by Stahl et al., can be used to interrogate the whole transcriptome coupled with spatial location, with most recent methods beginning to reach single-cell resolution. Combining single-cell transcriptomics with computational and spatial methodologies will increasingly yield data-rich cell-atlases that map the spatial location of all cellular gene activities at high resolution.

## Single-cell transcriptomics is transforming our understanding of health and disease

As mentioned above, single-cell transcriptomic approaches were first applied to model organism, and continue to be instrumental in driving forwards the understanding of cells and tissues in the mouse and other organisms such as zebrafish, worm etc. In 2018, two high-throughput compendia of murine tissues profiled by single-cell transcriptomics were published (e.g. refs. ^7,8^). These compendia provide data across many tissues using relatively uniform protocols, offering a rich biological resource. Focusing in on specific organs, a modified version of smFISH (osmFISH) was used to build an atlas of the mouse brain, giving a detailed view of cell identifty and exact position within the tissue^9^.

**Fig. 2:**
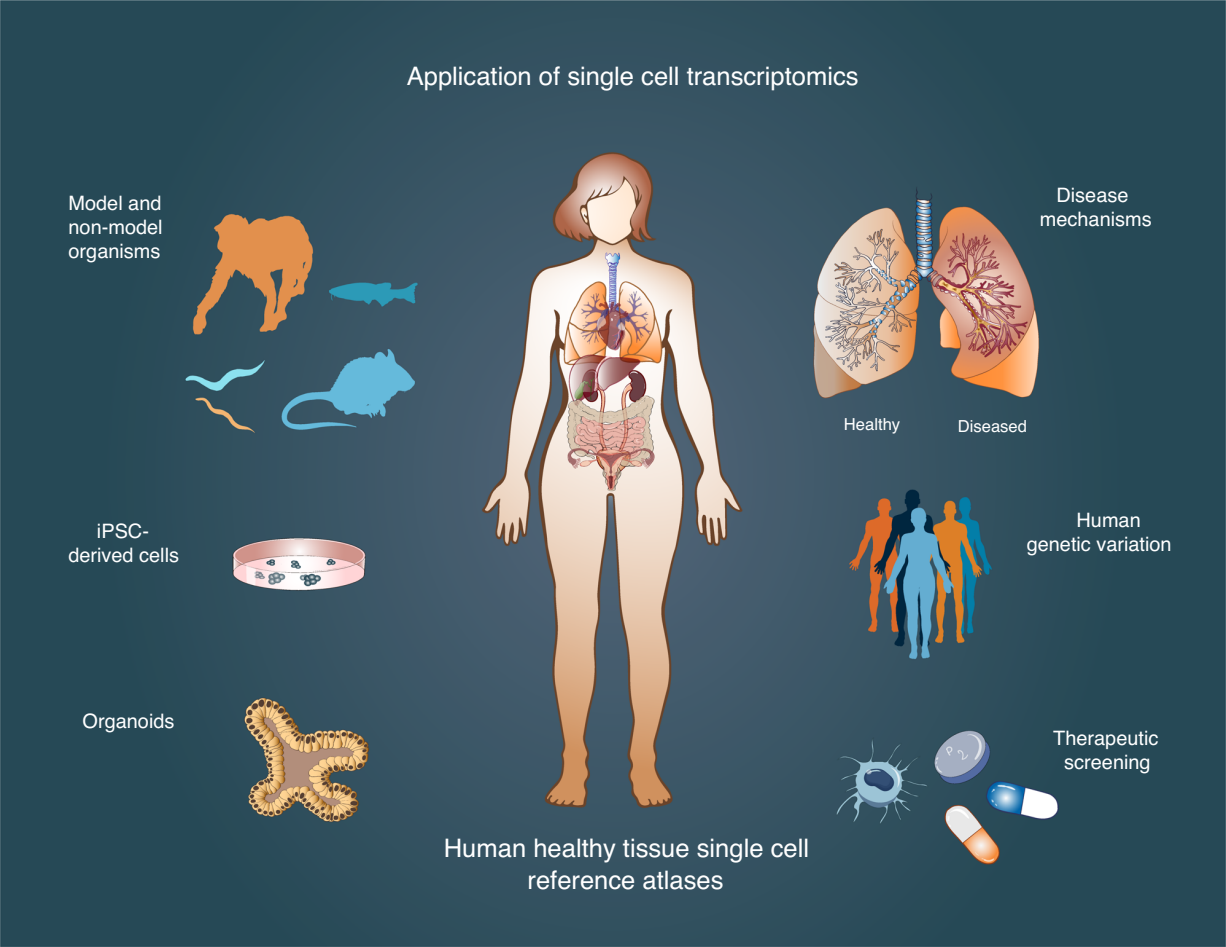
Application of single-cell technologies. Single-cell transcriptomics are being used to create reference maps of healthy human tissues, organs and systems at single-cell resolution. These approaches are also being applied to understand non-human organisms, including mice and non-human primates. Analysing healthy versus diseased tissues and genetic variation between individuals is also a valuable platform for the understanding of disease mechanisms. Factors identified from in vivo single-cell transcriptomic studies can also be applied to generate improved in vitro models and responses to therapeutic screening can be assayed at the single-cell level.

The scaling of single-cell technologies is ultimately applicable to the entire human body. Therefore, catalysed by this single-cell revolution, the Human Cell Atlas (HCA) global consortium was co-founded in 2016, led by one of us (Sarah Teichmann) and Aviv Regev (Broad Institute) and including colleagues excited by the vision. The HCA is using high-throughput technologies at single-cell resolution to map cells in human tissue, analogous to a “Google maps” of the human body. The value of high-resolution transcriptomic data of individual cells in their tissue context is profound and is generating fundamental knowledge of each human tissue studied by the HCA community. A healthy reference atlas of both human tissues and model organisms is a basic prerequisite to assessing changes in ageing, disease and in response to therapeutic treatment (Fig. 2).

Examples of new understanding into basic biology from cell atlasing of human tissues has been highlighted as especially powerful by studies of human development. The first single-cell atlas of the maternal-fetal interface in early pregnancy (first trimester placenta and decidua) revealed new cell states (e.g. NK and stromal cells), and a more detailed cellular and molecular understanding of the layered architecture of this tissue and its role in maternal immune tolerance of paternal antigens^10^. This study revealed mechanisms behind healthy pregnancy and increases our understanding of how these could change resulting in conditions such as pre-eclampsia.

Ground-breaking single-cell-atlases of development have been further extended to fundamental organs and systems including for example brain, heart, liver, thymus, gut and kidney. For example analysing thymic development at single-cell resolution has deepened understanding of new stromal and epithelial cell states and novel T cell types and indicates new principles of naïve T cell V(D)J repertoire formation, revealing that the human naïve T cell repertoire is biased in its receptor gene segments prior to pathogen exposure^11^.

Single-cell transcriptomics have been applied to a spectrum of diseases including cancer and auto-inflammatory disease. The airways are a good example of this, where comparison of healthy and asthmatic lung at single-cell level uncovered novel mucosal ciliated cell states, and CD4+ T cell states in health (tissue migratory CD4+ T cell) and disease (pathogenic Th2 cells)^12^. This revealed potential new therapeutic targets and provides a framework for the use of single-cell transcriptomics in target discovery. Use of scRNA-seq and murine lineage tracing have been used to analyse the cellular composition of the tracheal airway, revealing the cystic fibrosis gene CFTR is expressed in a rare ionocyte cell population at a higher level than in abundant ciliated cells^13^. Insights such as these are transforming our understanding of diseases with genetic components such as cystic fibrosis and asthma.

In recent months single-cell transcriptomics has been harnessed to further our biological understanding of COVID-19 infection in response to the current global pandemic. Single-cell expression analysis of the coronavirus receptor ACE2, viral entry associated protease TMPRSS2 and infection associated genes in healthy human individuals and non-human primates across a range of tissues gives insights into potential sites of viral transmission. Two specific cell types within the nose look likely to be sites of infection and could explain high rates of transmission (reviewed in ref. ^14^). This provides a valuable platform for investigating infection, transmission and developing clinical strategies for prevention and therapy with further single-cell studies underway.

## Future outlook

An exciting area is single-cell transcriptomics for improved engineering of in vitro cellular models such as organoids. It is becoming clear that single-cell transcriptomics can be applied across a breadth of medically important and competitive areas, including cell-based models, cell therapies, regenerative medicine and target discovery.

The utility of single-cell transcriptomics for in-depth validation of organoid models is demonstrated with single-cell RNA-seq applied to models of the dorsal forebrain, showing reliable recapitulation of the variety of cell types seen in the human cerebral cortex^15^. HCA data provides a high-dimensional description of cell states, including developmental pathways beginning from early first trimester human embryogenesis. This provides a precise template for in vitro models, and by comparing in vivo and in vitro data, we can predict the factors required to generate improved in vitro systems such as iPSC-derived cells and organoids. Insights from organ cell-atlases, exemplified by the precise characterisation of CFTR expressing cells in the lung, can be applied to analysis of gene expression in specific cell types to inform on the most accurate targeting of gene editing methodologies such as CRISPR-based editing.

As spatial transcriptomic technologies reach single-cell resolution they hold a great deal of promise for study of cells in tissues with complex morphology, such as regions of the brain and endometrium. The field is also tackling the challenge of single-cell spatial transcriptomics of whole organs and this will be hugely beneficial for the understanding of fundamental processes such as development. Increasing development of technologies compatible with FFPE tissue positions these methods as next generation diagnostic and pathology tools.

The surge of success in single-cell technologies has developed through the combination of academic research together with commercial initiatives to make robust, scalable and affordable technologies. Underpinning this has been the continual development of computational methods for data processing, analysis and integration. Happy tenth birthday single-cell transcriptomics! May the next decade be even more exciting than the last!
